# Supplementary material for: Hybrid Models and Biological Model Reduction with PyDSTool
Source: PLoS Comput Biol. 2012 Aug 9;8(8):e1002628. doi: 10.1371/journal.pcbi.1002628 (PMC3415397; doi:10.1371/journal.pcbi.1002628)
Supplement: Text S4 — Complete source code for the PyDSTool package (version 0.88.120504). Includes API documentation and help files linking to web pages. This file is identical to the current public release on Sourceforge.net. (ZIP) [file pcbi.1002628.s004.zip › PyDSTool/html/PyDSTool.parseUtils-module.html]

xml version="1.0" encoding="ascii"?


PyDSTool.parseUtils


| Home | Trees | Indices | Help | PyDSTool |
| --- | --- | --- | --- | --- |

Package PyDSTool ::
Module parseUtils

[hide private]

[frames] | no frames]

# Module parseUtils

source code

```
Parser utilities.

Robert Clewley, September 2005.

Includes AST code by Pearu Peterson and Ryan Gutenkunst,
  modified by R. Clewley.
```


|  |  |
| --- | --- |
| Classes | [hide private] |

symbolMapClass  
Abstract class for hassle-free symbol re-mappings.

auxfnDBclass  
Auxiliary function database, for use by parsers.

parserObject  
Alphanumeric symbol (pseudo-)parser for mathematical expressions.


|  |  |
| --- | --- |
| Functions | [hide private] |

|  |  |
| --- | --- |
| mapPowStr(t, p=`'``**``'`)  Input an expression of the form 'pow(x,y)'. | source code |

|  |  |
| --- | --- |
| toCircumflexSyntax(t) | source code |

|  |  |
| --- | --- |
| toDoubleStarSyntax(t) | source code |

|  |  |
| --- | --- |
| toPowSyntax(t) | source code |

|  |  |
| --- | --- |
| temp\_macro\_names(s) | source code |

|  |  |
| --- | --- |
| temp\_macro\_names\_inv(s) | source code |

|  |  |
| --- | --- |
| convertPowers(s, target=`'``pow``'`)  convertPowers takes a string argument and maps all occurrences of power sub-expressions into the chosen target syntax. | source code |

|  |  |
| --- | --- |
| ensureints(t)  Ensure that any floating point constants appearing in t that are round numbers get converted to integer representations. | source code |

|  |  |
| --- | --- |
| splitargs(da, lbraces=`[``'``(``'``]`, rbraces=`[``'``)``'``]`)  Function to split string-delimited arguments in a string without being fooled by those that occur in function calls. | source code |

|  |  |
| --- | --- |
| ast2shortlist(t) | source code |

|  |  |
| --- | --- |
| sym2name(t) | source code |

|  |  |
| --- | --- |
| string2ast(t) | source code |

|  |  |
| --- | --- |
| ast2string(t) | source code |

|  |  |
| --- | --- |
| splitastLR(t) | source code |

|  |  |
| --- | --- |
| dopower(l, r, pow\_str=`'``pow(%s,%s)``'`) | source code |

|  |  |
| --- | --- |
| domul(l, r) | source code |

|  |  |
| --- | --- |
| dodiv(l, r) | source code |

|  |  |
| --- | --- |
| doadd(l, r) | source code |

|  |  |
| --- | --- |
| dosub(l, r) | source code |

|  |  |
| --- | --- |
| doneg(l) | source code |

|  |  |
| --- | --- |
| ensuredecimalconst(t) | source code |

|  |  |
| --- | --- |
| trysimple(t, do\_decimal=False) | source code |

|  |  |
| --- | --- |
| ensureparen\_div(tt) | source code |

|  |  |
| --- | --- |
| ensureparen(t, flag=0, ismul=0, do\_decimal=False) | source code |

|  |  |
| --- | --- |
| ensurebare(t)  Ensure no braces in string expression (where possible). | source code |

|  |  |
| --- | --- |
| collect\_numbers(t)  Re-arrange 'term' or 'arith\_expr' expressions to combine numbers. | source code |

|  |  |
| --- | --- |
| simplify\_str(s) | source code |

|  |  |
| --- | --- |
| simplify(t) | source code |

|  |  |
| --- | --- |
| isToken(s, treatMultiRefs=False) | source code |

|  |  |
| --- | --- |
| isNameToken(s, treatMultiRefs=False) | source code |

|  |  |
| --- | --- |
| isIntegerToken(arg) | source code |

|  |  |
| --- | --- |
| isNumericToken(arg) | source code |

|  |  |
| --- | --- |
| findNumTailPos(s)  Find position of numeric tail in alphanumeric string. | source code |

|  |  |
| --- | --- |
| isHierarchicalName(s, sep=`'``.``'`, treatMultiRefs=False) | source code |

|  |  |
| --- | --- |
| isVectorClause(s) | source code |

|  |  |
| --- | --- |
| replaceSepList(speclist) | source code |

|  |  |
| --- | --- |
| replaceSepListInv(speclist) | source code |

|  |  |
| --- | --- |
| replaceSepInv(spec)  Invert default name separator replacement. | source code |

|  |  |
| --- | --- |
| replaceSep(spec, sourcesep=`'``.``'`, targetsep=`'``_``'`)  Replace hierarchy separator character with another and return spec string. | source code |

|  |  |
| --- | --- |
| mapNames(themap, target)  Map names in <target> argument using the symbolMapClass object <themap>, returning a renamed version of the target. | source code |

|  |  |
| --- | --- |
| replaceSepQSpec(spec, sourcesep, targetsep) | source code |

|  |  |
| --- | --- |
| replaceSepStr(spec, sourcesep, targetsep) | source code |

|  |  |
| --- | --- |
| joinStrs(strlist)  Join a list of strings into a single string (in order). | source code |

|  |  |
| --- | --- |
| joinAsStrs(objlist, sep=`'``'`)  Join a list of objects in their string representational form. | source code |

|  |  |
| --- | --- |
| count\_sep(specstr, sep=`'``,``'`)  Count number of specified separators (default = ',') in given string, avoiding occurrences of the separator inside nested braces | source code |

|  |  |
| --- | --- |
| parseMatrixStrToDictStr(specstr, specvars, m=0)  Convert string representation of m-by-n matrix into a single string, assuming a nested comma-delimited list representation in the input, and outputting a dictionary of the sub-lists, indexed by the ordered list of names specvars (specified as an argument). | source code |

|  |  |
| --- | --- |
| readArgs(argstr, lbchar=`'``(``'`, rbchar=`'``)``'`)  Parse arguments out of string beginning and ending with braces (default: round brace). | source code |

|  |  |
| --- | --- |
| findEndBrace(s, lbchar=`'``(``'`, rbchar=`'``)``'`)  Find position in string (or list of strings), s, at which final matching brace occurs (if at all). | source code |

|  |  |
| --- | --- |
| makeParList(objlist, prefix=`'``'`)  wrap objlist into a comma separated string of str(objects) | source code |

|  |  |
| --- | --- |
| wrapArgInCall(source, callfn, wrapL, wrapR=None, argnums=`[`0`]`, notFirst=False)  Add delimiters to single argument in function call. | source code |

|  |  |
| --- | --- |
| replaceCallsWithDummies(source, callfns, used\_dummies=None, notFirst=False)  Replace all function calls in source with dummy names, for the functions listed in callfns. | source code |

|  |  |
| --- | --- |
| addArgToCalls(source, callfns, arg, notFirst=`'``'`)  Add an argument to calls in source, to the functions listed in callfns. | source code |

|  |  |
| --- | --- |
| proper\_match(specstr, term)  Determine whether string argument 'term' appears one or more times in string argument 'specstr' as a proper symbol (not just as part of a longer symbol string or a number). | source code |

|  |  |
| --- | --- |
| remove\_indices\_from\_range(ixs, max\_ix)  From the indices 0:max\_ix+1, remove the individual index values in ixs. | source code |


|  |  |
| --- | --- |
| Variables | [hide private] |

all

predicate\_op

less

putmask

shape

complexfloating

DomainType

swapaxes

float32

int32

greater

float96

complex\_

ones

integer

API = `API_class()`

targetLangs = `['c', 'python', 'matlab']`

isfinite

API\_class

argmax

sign

Macheps

asarray

Continuous = `Continuous Domain`

intersect

null\_predicate = `null_predicate_class(None)`

complex64

atleast\_1d

Discrete = `Discrete Domain`

float64

float\_

less\_equal

os

float

array

log

complex128

zeros

ndarray

int\_

complex192

int0

int8

argmin

double

remain

norm

Inf

finfo

spy

clip

int

logical\_or

floating

any

null\_predicate\_class

NaN

sqrt

complex

take

LargestInt32 = `2147483647`

fit\_function

int16

mat

sys

npy

minpack

types

searchsorted

int64

exp

time

DO\_POW = `True`

DO\_DEC = `True`

protected\_scipynames = `['sign', 'mod']`

specialfns = `['airy', 'airye', 'ai_zeros', 'bi_zeros', 'ellipj...`

protected\_specialfns = `['special_airy', 'special_airye', 'spec...`

protected\_mathnames = `['acos', 'asin', 'atan', 'atan2', 'ceil'...`

protected\_randomnames = `['BPF', 'LOG4', 'NV_MAGICCONST', 'RECI...`

builtin\_auxnames = `['globalindepvar', 'initcond', 'heav', 'if'...`

protected\_macronames = `['for', 'if', 'max', 'min', 'sum']`

reserved\_keywords = `['and', 'not', 'or', 'del', 'for', 'if', '...`

convert\_power\_reserved\_keywords = `['del', 'for', 'if', 'is', '...`

protected\_allnames = `['acos', 'asin', 'atan', 'atan2', 'ceil',...`

builtinFnSigInfo = `{'for': 4, 'getbound': 2, 'getindex': 1, 'g...`

\_functions = `['readArgs', 'findEndBrace', 'makeParList', 'join...`

\_objects = `['protected_auxnamesDB', 'protected_allnames', 'pro...`

\_classes = `['symbolMapClass', 'parserObject', 'auxfnDBclass']`

\_constants = `['name_chars_RE', 'num_chars', 'ZEROS', 'ONES', '...`

\_symbfuncs = `['simplify', 'simplify_str', 'ensurebare', 'ensur...`

\_symbconsts = `['syms']`

name\_chars\_RE = `re.compile(r'\w')`

alphabet\_chars\_RE = `re.compile(r'[a-zA-Z0-9]')`

num\_chars = `['0', '1', '2', '3', '4', '5', '6', '7', '8', '9']`

POW\_STR = `'pow(%s,%s)'`

ZEROS = `['0', '0.0', '0.', '(0)', '(0.0)', '(0.)']`

ONES = `['1', '1.0', '1.', '(1)', '(1.0)', '(1.)']`

TENS = `['10', '10.0', '10.', '(10)', '(10.0)', '(10.)']`

NAMESEP = `'.'`

\_indentstr = `' '`

syms = `{0: 'ENDMARKER', 1: 'NAME', 2: 'NUMBER', 3: 'STRING', 4...`

protected\_auxnamesDB = `ModelSpec internal helper class: auxfnD...`

\_all\_complex = `(<type 'complex'>, <type 'numpy.complexfloating...`

\_all\_float = `(<type 'float'>, <type 'numpy.floating'>, <type '...`

\_all\_int = `(<type 'int'>, <type 'numpy.integer'>, <type 'numpy...`

\_all\_numpy\_complex = `(<type 'numpy.complex128'>, <type 'numpy....`

\_all\_numpy\_float = `(<type 'numpy.float64'>, <type 'numpy.float...`

\_all\_numpy\_int = `(<type 'numpy.int32'>, <type 'numpy.int32'>, ...`

\_complex\_types = `(<type 'complex'>, <type 'numpy.complexfloati...`

\_float\_types = `(<type 'float'>, <type 'numpy.floating'>)`

\_int\_types = `(<type 'int'>, <type 'numpy.integer'>)`

\_num\_equivtype = `{<type 'float'>: <type 'numpy.float64'>, <typ...`

\_num\_maxmin = `{<type 'numpy.int32'>: [-2147483648, 2147483647]...`

\_num\_name2equivtypes = `{'float': (<type 'float'>, <type 'numpy...`

\_num\_name2type = `{'float': <type 'numpy.float64'>, 'int': <typ...`

\_num\_type2name = `{<type 'float'>: 'float', <type 'int'>: 'int'...`

\_num\_types = `(<type 'float'>, <type 'int'>, <type 'numpy.float...`

\_pytypefromtype = `{<type 'numpy.int32'>: <type 'int'>, <type '...`

\_real\_types = `(<type 'int'>, <type 'numpy.integer'>, <type 'fl...`

\_seq\_types = `(<type 'list'>, <type 'tuple'>, <type 'numpy.ndar...`

s = `339`


|  |  |
| --- | --- |
| Function Details | [hide private] |

|  |  |
| --- | --- |
| mapPowStr(t, p=`'``**``'`) | source code |

Input an expression of the form 'pow(x,y)'. Outputs an expression of
the form x\*\*y, x^y, or pow(x,y) and where x and y have also been
processed to the target power syntax. Written by R. Clewley

|  |  |
| --- | --- |
| convertPowers(s, target=`'``pow``'`) | source code |

convertPowers takes a string argument and maps all occurrences of
power sub-expressions into the chosen target syntax. That option is one
of "\*\*", "^", or "pow".

|  |  |
| --- | --- |
| splitargs(da, lbraces=`[``'``(``'``]`, rbraces=`[``'``)``'``]`) | source code |

Function to split string-delimited arguments in a string without being
fooled by those that occur in function calls. Written by Pearu Peterson.
Adapted by Rob Clewley to accept different braces.

|  |  |
| --- | --- |
| ensurebare(t) | source code |

Ensure no braces in string expression (where possible). Written by
Robert Clewley

|  |  |
| --- | --- |
| collect\_numbers(t) | source code |

Re-arrange 'term' or 'arith\_expr' expressions to combine numbers.
Numbers go first unless in a sum the numeric term is negative and not all
the remaining terms are negative.

|  |  |
| --- | --- |
| findNumTailPos(s) | source code |

Find position of numeric tail in alphanumeric string.

e.g. findNumTailPos('abc678') = 3

|  |  |
| --- | --- |
| replaceSep(spec, sourcesep=`'``.``'`, targetsep=`'``_``'`) | source code |

Replace hierarchy separator character with another and return spec
string. e.g. "." -> "\_" Only replaces the
character between name tokens, not between numbers.

|  |  |
| --- | --- |
| mapNames(themap, target) | source code |

Map names in <target> argument using the symbolMapClass object
<themap>, returning a renamed version of the target. N.B. Only maps
the keys of a dictionary type

|  |  |
| --- | --- |
| readArgs(argstr, lbchar=`'``(``'`, rbchar=`'``)``'`) | source code |

Parse arguments out of string beginning and ending with braces
(default: round brace).

Returns a triple: [success\_boolean, list of arguments, number of
args]

|  |  |
| --- | --- |
| findEndBrace(s, lbchar=`'``(``'`, rbchar=`'``)``'`) | source code |

Find position in string (or list of strings), s, at which final
matching brace occurs (if at all). If not found, returns None.

s[0] must be the left brace character. Default left and right braces
are '(' and ')'. Change them with the optional second and third
arguments.

|  |  |
| --- | --- |
| replaceCallsWithDummies(source, callfns, used\_dummies=None, notFirst=False) | source code |

Replace all function calls in source with dummy names, for the
functions listed in callfns. Returns a pair (new\_source, d) where d is a
dict mapping the dummy names used to the function calls.

|  |  |
| --- | --- |
| remove\_indices\_from\_range(ixs, max\_ix) | source code |

From the indices 0:max\_ix+1, remove the individual index values in
ixs. Returns the remaining ranges of indices and singletons.

  


|  |  |
| --- | --- |
| Variables Details | [hide private] |

### specialfns

Value:
:   ```
    ['airy',
     'airye',
     'ai_zeros',
     'bi_zeros',
     'ellipj',
     'ellipk',
     'ellipkinc',
     'ellipe',
    ...
    ```

### protected\_specialfns

Value:
:   ```
    ['special_airy',
     'special_airye',
     'special_ai_zeros',
     'special_bi_zeros',
     'special_ellipj',
     'special_ellipk',
     'special_ellipkinc',
     'special_ellipe',
    ...
    ```

### protected\_mathnames

Value:
:   ```
    ['acos',
     'asin',
     'atan',
     'atan2',
     'ceil',
     'cos',
     'cosh',
     'degrees',
    ...
    ```

### protected\_randomnames

Value:
:   ```
    ['BPF',
     'LOG4',
     'NV_MAGICCONST',
     'RECIP_BPF',
     'Random',
     'SG_MAGICCONST',
     'SystemRandom',
     'TWOPI',
    ...
    ```

### builtin\_auxnames

Value:
:   ```
    ['globalindepvar', 'initcond', 'heav', 'if', 'getindex', 'getbound']
    ```

### reserved\_keywords

Value:
:   ```
    ['and',
     'not',
     'or',
     'del',
     'for',
     'if',
     'is',
     'raise',
    ...
    ```

### convert\_power\_reserved\_keywords

Value:
:   ```
    ['del',
     'for',
     'if',
     'is',
     'raise',
     'assert',
     'elif',
     'from',
    ...
    ```

### protected\_allnames

Value:
:   ```
    ['acos',
     'asin',
     'atan',
     'atan2',
     'ceil',
     'cos',
     'cosh',
     'degrees',
    ...
    ```

### builtinFnSigInfo

Value:
:   ```
    {'for': 4,
     'getbound': 2,
     'getindex': 1,
     'globalindepvar': 1,
     'heav': 1,
     'if': 3,
     'initcond': 1,
     'max': 1,
    ...
    ```

### \_functions

Value:
:   ```
    ['readArgs',
     'findEndBrace',
     'makeParList',
     'joinStrs',
     'parseMatrixStrToDictStr',
     'joinAsStrs',
     'replaceSep',
     'wrapArgInCall',
    ...
    ```

### \_objects

Value:
:   ```
    ['protected_auxnamesDB',
     'protected_allnames',
     'protected_macronames',
     'protected_mathnames',
     'protected_randomnames',
     'builtin_auxnames',
     'protected_scipynames',
     'protected_specialfns',
    ...
    ```

### \_constants

Value:
:   ```
    ['name_chars_RE',
     'num_chars',
     'ZEROS',
     'ONES',
     'NAMESEP',
     '_indentstr']
    ```

### \_symbfuncs

Value:
:   ```
    ['simplify',
     'simplify_str',
     'ensurebare',
     'ensureparen',
     'trysimple',
     'ensuredecimalconst',
     'doneg',
     'dosub',
    ...
    ```

### syms

Value:
:   ```
    {0: 'ENDMARKER',
     1: 'NAME',
     2: 'NUMBER',
     3: 'STRING',
     4: 'NEWLINE',
     5: 'INDENT',
     6: 'DEDENT',
     7: 'LPAR',
    ...
    ```

### protected\_auxnamesDB

Value:
:   ```
    ModelSpec internal helper class: auxfnDBclass object
    ```

### \_all\_complex

Value:
:   ```
    (<type 'complex'>,
     <type 'numpy.complexfloating'>,
     <type 'numpy.complex128'>,
     <type 'numpy.complex64'>,
     <type 'numpy.complex128'>)
    ```

### \_all\_float

Value:
:   ```
    (<type 'float'>,
     <type 'numpy.floating'>,
     <type 'numpy.float64'>,
     <type 'numpy.float32'>,
     <type 'numpy.float64'>)
    ```

### \_all\_int

Value:
:   ```
    (<type 'int'>,
     <type 'numpy.integer'>,
     <type 'numpy.int32'>,
     <type 'numpy.int32'>,
     <type 'numpy.int8'>,
     <type 'numpy.int16'>,
     <type 'numpy.int32'>,
     <type 'numpy.int64'>)
    ```

### \_all\_numpy\_complex

Value:
:   ```
    (<type 'numpy.complex128'>,
     <type 'numpy.complex64'>,
     <type 'numpy.complex128'>)
    ```

### \_all\_numpy\_float

Value:
:   ```
    (<type 'numpy.float64'>,
     <type 'numpy.float32'>,
     <type 'numpy.float64'>)
    ```

### \_all\_numpy\_int

Value:
:   ```
    (<type 'numpy.int32'>,
     <type 'numpy.int32'>,
     <type 'numpy.int8'>,
     <type 'numpy.int16'>,
     <type 'numpy.int32'>,
     <type 'numpy.int64'>)
    ```

### \_complex\_types

Value:
:   ```
    (<type 'complex'>, <type 'numpy.complexfloating'>)
    ```

### \_num\_equivtype

Value:
:   ```
    {<type 'float'>: <type 'numpy.float64'>,
     <type 'int'>: <type 'numpy.int32'>,
     <type 'numpy.integer'>: <type 'numpy.int32'>,
     <type 'numpy.floating'>: <type 'numpy.float64'>,
     <type 'numpy.int8'>: <type 'numpy.int32'>,
     <type 'numpy.int16'>: <type 'numpy.int32'>,
     <type 'numpy.int32'>: <type 'numpy.int32'>,
     <type 'numpy.int32'>: <type 'numpy.int32'>,
    ...
    ```

### \_num\_maxmin

Value:
:   ```
    {<type 'numpy.int32'>: [-2147483648, 2147483647],
     <type 'numpy.float64'>: [-inf, inf]}
    ```

### \_num\_name2equivtypes

Value:
:   ```
    {'float': (<type 'float'>,
               <type 'numpy.floating'>,
               <type 'numpy.float64'>,
               <type 'numpy.float32'>,
               <type 'numpy.float64'>),
     'int': (<type 'int'>,
             <type 'numpy.integer'>,
             <type 'numpy.int32'>,
    ...
    ```

### \_num\_name2type

Value:
:   ```
    {'float': <type 'numpy.float64'>, 'int': <type 'numpy.int32'>}
    ```

### \_num\_type2name

Value:
:   ```
    {<type 'float'>: 'float',
     <type 'int'>: 'int',
     <type 'numpy.integer'>: 'int',
     <type 'numpy.floating'>: 'float',
     <type 'numpy.int8'>: 'int',
     <type 'numpy.int16'>: 'int',
     <type 'numpy.int32'>: 'int',
     <type 'numpy.int32'>: 'int',
    ...
    ```

### \_num\_types

Value:
:   ```
    (<type 'float'>,
     <type 'int'>,
     <type 'numpy.floating'>,
     <type 'numpy.integer'>)
    ```

### \_pytypefromtype

Value:
:   ```
    {<type 'numpy.int32'>: <type 'int'>,
     <type 'numpy.float64'>: <type 'float'>}
    ```

### \_real\_types

Value:
:   ```
    (<type 'int'>,
     <type 'numpy.integer'>,
     <type 'float'>,
     <type 'numpy.floating'>)
    ```

### \_seq\_types

Value:
:   ```
    (<type 'list'>, <type 'tuple'>, <type 'numpy.ndarray'>)
    ```

  


| Home | Trees | Indices | Help | PyDSTool |
| --- | --- | --- | --- | --- |

|  |  |
| --- | --- |
| Generated by Epydoc 3.0.1 on Fri May 4 15:24:06 2012 | http://epydoc.sourceforge.net |
